# Zoo: Selecting Transcriptomic and Methylomic Biomarkers by Ensembling Animal-Inspired Swarm Intelligence Feature Selection Algorithms

**DOI:** 10.3390/genes12111814

**Published:** 2021-11-18

**Authors:** Yuanyuan Han, Lan Huang, Fengfeng Zhou

**Affiliations:** Key Laboratory of Symbolic Computation and Knowledge Engineering of Ministry of Education, College of Computer Science and Technology, Jilin University, Changchun 130012, China; hanyy19@mails.jlu.edu.cn (Y.H.); huanglan@jlu.edu.cn (L.H.)

**Keywords:** feature selection, swarm intelligence, machine learning, prediction, program code

## Abstract

Biological omics data such as transcriptomes and methylomes have the inherent “large p small n” paradigm, i.e., the number of features is much larger than that of the samples. A feature selection (FS) algorithm selects a subset of the transcriptomic or methylomic biomarkers in order to build a better prediction model. The hidden patterns in the FS solution space make it challenging to achieve a feature subset with satisfying prediction performances. Swarm intelligence (SI) algorithms mimic the target searching behaviors of various animals and have demonstrated promising capabilities in selecting features with good machine learning performances. Our study revealed that different SI-based feature selection algorithms contributed complementary searching capabilities in the FS solution space, and their collaboration generated a better feature subset than the individual SI feature selection algorithms. Nine SI-based feature selection algorithms were integrated to vote for the selected features, which were further refined by the dynamic recursive feature elimination framework. In most cases, the proposed Zoo algorithm outperformed the existing feature selection algorithms on transcriptomics and methylomics datasets.

## 1. Introduction

The accelerated accumulation of omics data has been benefited by the rapid innovation and development of various high-throughput omics technologies [1]. There are many types of omics data, including genomics data, transcriptomics data, methylomics data, metabolomics data and proteomics data, that describe the biological systems from different perspectives [2]. They also introduce the challenge of high feature dimensionalities for data analysis, i.e., the number of features in a dataset usually far exceeds that of samples [3]. This data dimension disaster may be partly solved by dimension reduction [4] or feature selection [5,6].

Feature selection is an NP-hard problem whose global optimal solution cannot be found within polynomial time [7]. Thus, except for the exhaustive searching strategy, all the existing feature selection algorithms try to find feature subsets with locally optimized performances. Feature selection algorithms may be roughly grouped as filters and wrappers [8]. A filter ranks the features in the descending order of their associations with the class labels, and the association between a feature and the class label may be measured by various metrics such as the *t*-test [9] and correlation coefficient [10]. A wrapper iteratively evaluates a heuristically generated feature subset by a predefined classifier and outputs the feature subset with the best optimization performance [11,12]. More complicated frameworks have also been designed to find feature subsets with better prediction performances, e.g., embedded [13] and meta-heuristic [14] feature selection algorithms.

Swarm intelligence (SI) is a type of meta-heuristic feature selection algorithm that imitates living organisms’ behaviors to generate intermediate feature subsets for performance evaluations [15]. An SI feature selection algorithm extracts the living organisms’ behaviors as abstract algorithmic operations for feature subsets, including genetic information exchanges and dynamic searching strategies [16]. Popular SI feature selection algorithms include Grey Wolf Optimization (GWO) [17], Cuckoo Searching (CS) [18], the Whale Optimization Algorithm (WOA) [19], the bat algorithm (BA) [20], the Firefly Algorithm (FA) [21], the moth–flame optimization algorithm (MFO) [22], Particle Swarm Optimization (PSO) [23,24], the Manta Rays Foraging Optimization algorithm (MRFO) [25] and the Dragonfly algorithm (DF) [26]. Datasets have inherent patterns and an SI algorithm usually cannot guarantee the choice of the best feature subset on all the datasets. Rostami et al. studied 11 types of state-of-the-art swarm intelligence for feature selection problems. The results showed that swarm intelligence algorithms tend to fall into local optimal solutions for high-dimensional data sets, and different swarm intelligence algorithms perform differently [27]. Brezocnik et al., found that promising swarm intelligence algorithms used in feature selection algorithms included PSO, BA, GWO, FA, DA and ant colony optimization (ACO), and many swarm intelligence algorithms were rarely applied to feature selection problems. Some of the latest algorithms, such as BCO, CS, FA and GWO, were also used in conjunction with other techniques and showed very promising results in FS [28].

Our study revealed that the integration of multiple SI feature selection algorithms might deliver satisfying solutions for most datasets. Thus, this study integrated and evaluated the recommended feature subsets of nine SI-based feature selection algorithms, including WOA, BA CS, FA, MFO, PSO, MRFO, DF and GWO. A majority voting strategy was used to find the features recommended by more than four SI feature selection algorithms, and the redundant features were further refined by the dynamical recursive feature elimination (dRFE) strategy [29]. The proposed feature selection algorithm Zoo was comprehensively evaluated for the prediction performances of its recommended feature subsets, and its source code is publicly available at http://www.healthinformaticslab.org/supp/resources.php (accessed on 9 November 2021).

## 2. Materials and Methods

### 2.1. Summary of Datasets

This study evaluated the performances of feature selection algorithms using the binary classification problems of transcriptome and methylome datasets, as shown in Table S1. Firstly, the proposed swarm intelligent (SI) feature selection algorithm Zoo was tuned using 17 popular transcriptome datasets [30], consisting of 15 cancer datasets and 2 cardiovascular disease ones. Seventeen publicly available transcriptome datasets were used for algorithm tuning. They include the 5 datasets of Myeloma (accession: GDS531) [31], Gastric (accession: GSE37023) [32], Gastric1/Gastric2 (accession: GSE29272) [33], T1D (accession: GSE35725) [34] and Stroke (accession: GSE22255) [35] obtained from the NCBI Gene Expression Omnibus (GEO) database; the 6 datasets of DLBCL [36], Prostate [37], ALL [38], CNS [39], Lymphoma [40] and Adenoma [41] provided from the Broad Institute Genome Data Analysis Center; and the 2 datasets of Colon [42] and Leukemia [43] found from R/Bioconductor packages colonCA and golubEsets, respectively. The ALL dataset was divided into 4 datasets: ALL1, ALL2, AL3 and ALL4, according to different phenotypic annotations.

Ten additional transcriptome datasets were chosen in order to compare the prediction performances of the proposed algorithm Zoo and the existing feature selection algorithms, as shown in Table S1. These ten binary classification datasets were retrieved from the Gene Expression Omnibus (GEO) database [44]. The thyroid cancer samples with different phenotypes (GSE35570-1 and GSE35570-2, under the accession number GSE35570) were profiled using the platform GPL570 (HG-U133_Plus_2) Affymetrix Human Genome U133 Plus 2.0 Array), which has 54,675 features. This GPL570 platform was also used to profile the transcriptomes of peripheral blood lymphocytes with and without autism (GSE25507) [45], Parkinson’s disease and controls (GSE99039) [46], metastatic recurrent and primary colorectal cancers (GSE21510) [47], lung cancers and the matched distant normal lung tissues (GSE33532) [48], female lung cancers and controls (GSE19804) [49], breast cancers and controls (GSE27562) [50] and lung cancers in early and late stages (GSE30219) [51]. The transcriptomes of lung cancers in males and females (GSE4824) [52] were profiled using another platform, GPL96 (HG-U133A) Affymetrix Human Genome U133A Array), which has 22,283 features.

Five methylome datasets were chosen to evaluate how the investigated feature selection algorithms perform on different types of omics data, as shown in Table S1. The methylation platform GPL13534 (Illumina HumanMethylation450 BeadChip, HumanMethylation450_15017482) was used to profile the methylomes of these 5 datasets, which provided 485,577 methylation features. This study abstracted binary classification problems from the methylomes of peripheral blood mononuclear cells for smokers and non-smokers (GSE53045) [53], breast cancers and normal samples (GSE66695) [54], normal fallopian tube samples with and without BRCA1/2 mutations (GSE74845) [55], Alzheimer’s disease and controls (GSE80970) [56] and gastric light or mild intestinal metaplasia (GSE103186) [57]. Features with missing data were removed from further analysis.

A stratified split strategy of the ratios 1:1:1 was used to divide each dataset into the training, validation and testing subsets. The features were selected based on the training dataset, and the parameters were optimized based on the validation dataset. The final performance was calculated using the test dataset.

### 2.2. Performance Metrics

This study evaluated a feature selection algorithm according to the binary classification performances of its recommended feature subset. A binary classification problem had two classes of samples, i.e., positive and negative ones. The numbers of positive and negative samples were denoted as *P* and *N* [58]. The prediction accuracy of the positive samples was calculated as sensitivity, i.e., *Sn* = *TP*/(*TP* + *FN*), where *TP* and *FN* were the numbers of correctly and incorrectly predicted positive samples, respectively. The specificity (*Sp*) was similarly defined for the negative samples, and *Sp* = *TN*/(*TN* + *FP*), where *TN* and *FP* were the numbers of true negatives and false positives, respectively. The overall accuracy was defined as *Acc* = (*TP* + *TN*)/(*TP* + *FN* + *TN* + *FP*). The metric *Acc* was used to evaluate all the feature selection algorithms.

### 2.3. Stratified k-Fold Cross Validation Strategy

A stratified three-fold cross-validation (S3FCV) strategy [59] was utilized to evaluate the classification performances. The random seed was set to 0. S3FCV randomly split the positive and negative samples into three equally sized subsets. In each iteration, one positive and one negative subset was combined as the test set, and the remaining samples were used to train the classification model. S3FCV ensured that each sample was used as a test sample once and only once, and the same ratio between positive and negative samples was maintained in the training and test datasets. This study implemented and carried out all the experiments in the Python programming language version 3.7.6.

### 2.4. Nine Swarm Intelligence Feature Selection Algorithms

Swarm intelligence (SI) optimization algorithms have demonstrated powerful capabilities in many combinatorial optimization problems, and many SI algorithms have been modified for the feature selection task [27,60].

The Whale Optimization Algorithm (WOA) mimics the hunting behavior of humpback whales [19,61] by the bubble-net feeding method. WOA randomly searches for solutions in the exploration stage, and the exploitation stage carries out a delicate local search in the search space around a promising solution revealed in the exploration stage. WOA uses a logarithmic spiral function to mathematically formulate the behavior whereby a humpback whale creates a spiral bubble net around the prey.

The Bat Algorithm (BA) carries out its optimization procedure using operations inspired by the bat’s echolocation behaviors [62]. A bat’s flight is affected by the echolocation’s frequency, speed and loudness, and these variables are adjusted based on the proximity to the target.

Cuckoo Search (CS) searches for the optimization target using three rules inspired by the brood parasitism of certain species of cuckoos [63,64]. CS assumes that each cuckoo lays an egg in one randomly selected nest; the best place among the selected nests will be reserved for the next generation of cuckoos, and the number of available bird nests is fixed. The host bird of a nest has a probability of finding the cuckoo egg in its nest. If this happens, the host bird will remove the cuckoo egg, or build a new nest instead.

Yang X. S. developed the Firefly Algorithm (FA) in 2008 by mimicking the behaviors of firefly flashing characteristics [65,66]. Fireflies are unisex, and a firefly with a brighter flashing light attracts neighboring fireflies to move toward it.

The Moth–Flame Optimization (MFO) algorithm is a meta-heuristic algorithm simulating the navigation mode of moths [22,67]. A moth executes a straight-line flight to a remote target by maintaining a fixed angle with the moon in the night. This habit causes moths to be trapped spirally around artificial lights. MFO mathematically formulates this behavior to optimize the feature selection procedure.

Particle Swarm Optimization (PSO) places a swarm of particles in the solution space and evaluates the fitness of each particle [68,69]. The movement of each particle will be defined by its own history locations, the best locations and the other particles’ information. Random perturbations will also be considered. The whole swarm is expected to move close to a locally optimal solution in regard to the fitness function.

The Manta Ray Foraging Optimization (MRFO) mathematically formulates the foraging strategy of manta rays [25,70]. Three foraging strategies of manta rays are abstracted as optimization rules, i.e., chain foraging, cyclone foraging and somersault foraging.

The Dragonfly Algorithm (DF) is another popular optimization algorithm inspired by the foraging and migration behaviors of dragonflies [26,71]. The operation separation mimics the mechanism whereby two neighboring dragonflies avoid collisions with each other. The second operation alignment models when the dragonflies match their movement velocities with neighboring ones. The last operation cohesion models the dragonflies’ tendency toward the neighborhood’s mass center.

Grey Wolf Optimization (GWO) is a bio-inspired SI optimization algorithm that mimics the hunting process of grey wolves in nature [72,73]. A wolf pack consists of four levels of social hierarchies, i.e., alpha, beta, omega and delta wolves. The alpha wolves make decisions, and the betas assist the alphas in decision making. The deltas are minors to alphas and betas and are responsible for scouting and hunting, while the omegas have the lowest priority in eating the preys. The best feature selection solution is defined as the alpha, while the second and third best solutions are beta and delta. The rest of the solutions are the omega wolves. The next generation of wolves is updated using the combined information of alpha, beta, delta and the random information.

### 2.5. The Ensemble SI-Based Feature Selection Algorithm Zoo

**Figure 1:**
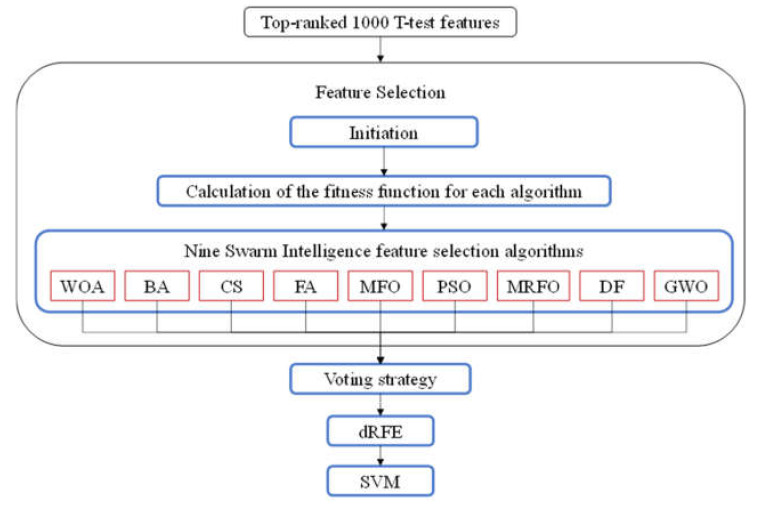
Flowchart of the proposed feature selection algorithm Zoo.

The first step of the proposed Zoo algorithm evaluated the association of each feature with the class label in the training dataset using the *t*-test, and ranked the features in ascending order of the *t*-test *p*-values, as shown in Figure 1. Most swarm intelligence (SI) algorithms had high time complexities due to the population-based random solution searching strategy. In order to avoid an extremely long running time, this study retrieved the top-ranked 1000 features to evaluate the SI algorithms.

Secondly, the 9 SI feature selection algorithms in the above section were applied to the datasets using the selected 1000 features. The binary version of each SI algorithm was used as a feature selection algorithm in this study. Ten random runs of each SI algorithm were carried out, and the feature subset with the highest prediction accuracy on the validation dataset was output as the final solution.

Thirdly, each feature was counted by its vote by the nine SI feature selection algorithms, and the majority rule was used to generate the subset of features. A dynamic recursive feature elimination (dRFE) strategy was used to further refine the subset of features The S3FCV strategy was used in the SVM-based dRFE framework with 7 as the maximal number of features removed in each iteration. The feature subset achieving the best prediction accuracy was delivered as the final output.

### 2.6. Binary Animal-Inspired SI-Based Feature Selection Algorithms

Feature selection may be formulated as a binary SI algorithm, in which a binary-valued array represents a feature subset, and the value 1 or 0 in each position of this array denotes the choice or not of a corresponding feature. All of the nine animal-inspired SI algorithms investigated in this study are equation-based algorithms [74], and they randomly initiate a set of feature subsets for their own optimization procedures.

The binary versions of the Manta Ray Foraging Optimization (MRFO) were re-implemented using the Python code from the Matlab code [72]. Additionally, the Dragonfly Algorithm (DF) was implemented based on the original Matlab codes. The binary feature selection algorithms of the other seven SI algorithms were implemented using the open-source framework Evolopy-FS [75,76].

The fitness function is defined so as to integrate the effects of both classification error rate and the number of selected features, similar to [77].

(1) $$Fitness=\omega E+\left(1-\omega \right)Selected/Dimension\;$$

The parameter *ω* is used to balance the two factors of the error rate *E* and the rate of selected features *Selected*/*Dimension*, where Selected and Dimension are the numbers of selected and all the features. This study set *ω* = 0.9.

Three classifiers were used to calculate the classification performances of the fitness functions using the training and testing subsets split by the ratio 2:1 of the training dataset. The three classifiers are Support Vector Machine (SVM), Naïve Bayes (NBayes) and k Nearest Neighbor (KNN).

### 2.7. The Existing Feature Selection Algorithms

The proposed Zoo algorithm was compared with nine existing feature selection algorithms using three binary classifiers. In order to maintain a fair comparison, the number of features selected by a feature selection algorithm was set to be the same as Zoo. The parameters of the nine feature selection algorithms for comparison are described in Table S2. Each algorithm is abbreviated in the brackets and referenced as a function in the Python package sklearn version 0.19.2. The features may be ranked by four algorithms, i.e., adaptive boosting (AdaBoost), the gini index of the decision tree classifier (DT_gini), Gradient Boosting (GB) and Random Forest (RF). A binary classification model was trained using one of two algorithms, i.e., L1 regularized logistic regression (LR_L1) and Linear Support Vector Machine (lSVC_L1). The model coefficients are used to rank the features in descending order. The Recursive Feature Elimination (RFE) strategy may be used with the two classifiers: Support Vector Machine (RFE_SVC) and Random Forest (RFE_RF). The function SelectKBest() was also used to select the top-ranked k features (abbreviated as SK_mic).

The performance metric maximum accuracy (mAcc) was used to evaluate the feature selection algorithm. The S3FCV strategy was used to calculate the classification performances using the five classifiers, i.e., logistic regression (LR), k Nearest Neighbor (KNN), Gaussian Naïve Bayes classifier (NBayes), Decision Tree (DT) and Support Vector Machine (SVM).

## 3. Results

### 3.1. Evaluating the Classifiers for the Selected Features

**Figure 2:**
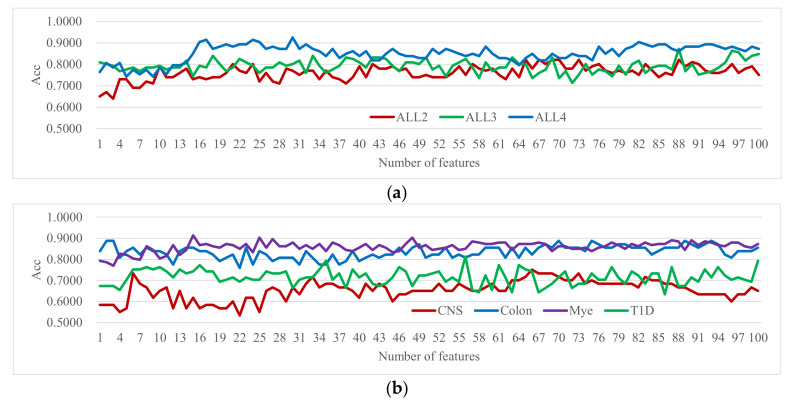
Incremental feature selection based on *t*-test for seven datasets. The vertical axis gives the accuracy (Acc) of the top-ranked k features by the SVM classifier. The horizontal axis lists the value of k. Acc was calculated using the S10FCV strategy. (**a**) The performance evaluation on the three leukemia datasets: ALL2, ALL3 and ALL4. (**b**) The performance evaluation on the four datasets: CNS, Colon, Mye and T1D.

Seven among the first seventeen transcriptome datasets received the worst prediction performances in the previous study [30], and these datasets were used to tune the algorithmic parameters in this study. The details of these datasets are annotated in Table S2. Figure 2 showed the experimental results of the *t*-test-based Incremental Feature Selection (IFS) strategies [78] with at most 100 features. The SVM classifier only achieved Acc = 0.7500 using 66 features for the dataset CNS. The best accuracy was only 0.9247 using 30 features for the dataset ALL4. Thus, these datasets need to be improved by finding better features for the prediction tasks and will be used in the following sections to tune the parameters.

**Figure 3:**
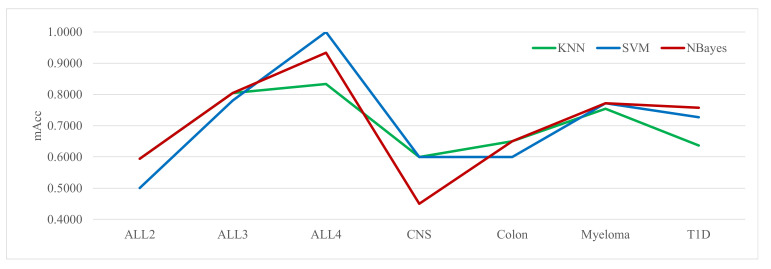
Performance comparison of the three classifiers for their integration in the fitness function of the Zoo feature selection algorithm. The horizontal axis lists the seven datasets, and the vertical axis gives the data of the performance metric mAcc using the S3FCV strategy. The metric mAcc was calculated as the maximum Acc using the five classifiers: NBayes, SVM, LR, DT and KNN on the Zoo-recommended features.

Three classifiers, SVM, NBayes and KNN, were evaluated for their classification performances when each was used in the fitness function of the Zoo feature selection algorithm, as shown in Figure 3. The fitness function was defined as *Fitness* = *ω* × *E* + (1 − *ω*) × *R*, where *E* was the error rate of the classification model, and *R* was the ratio of the selected features among all of them. This study set *ω* = 0.9.

The population size and the maximum number of iterations were set to 50 and 100 for all of the nine SI feature selection algorithms. The major parameters of the nine SI feature selection algorithms were set to the default values, as listed in Table S3. Each dataset was filtered by the *t*-test, and the top-ranked 1000 features were screened by a random run of each of the nine SI feature selection algorithms. A majority voting strategy was used to find the features recommended by more than four out of the nine SI feature selection algorithms. A further refining step using the dRFE algorithm was carried out to remove potentially redundant features in each feature subset. The remaining features were used to build the prediction model using the same classifier integrated in the fitness function.

Figure 3 shows that the classifier NBayes achieved the best classification performances for five out of the seven datasets, while the classifiers KNN and SVM performed the best only for four and three datasets, respectively. Thus, NBayes was used as the classifier integrated into the fitness function of the Zoo algorithm.

### 3.2. Finding the Best Population Size for Five SI Algorithms

**Figure 4:**
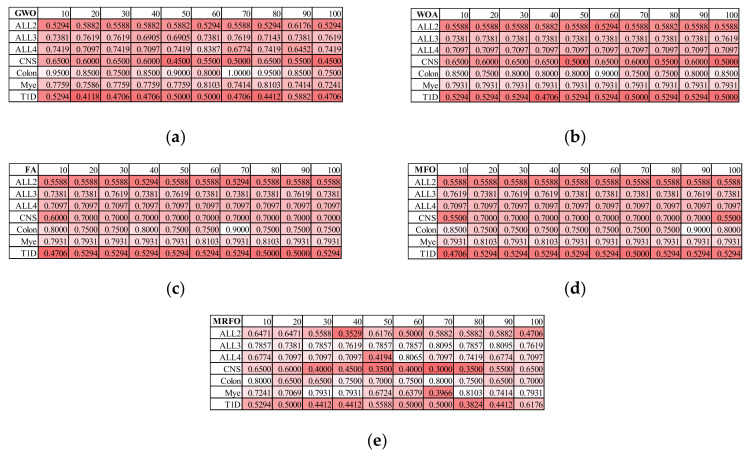
Evaluation of population sizes for the five SI feature selection algorithms. The prediction accuracies of the default classifier NBayes using the features recommended by the SI feature selection algorithms (**a**) GWO, (**b**) WOA, (**c**) FA, (**d**) MFO and (**e**) MRFO. The rows give the data for the Scheme 10. 20, 30, 40, 50, 60, 70, 80, 90 and 100. Each classification accuracy was colored by the red scale, with a deeper red color for a smaller accuracy.

The internal parameters of the five SI feature selection algorithms GWO/WOA/FA/MFO/MRFO were randomly generated, and their population sizes (variable N) were evaluated for the classification accuracies of their recommended features, as shown in Figure 4. Due to the high time complexities of the SI algorithms, all the seven datasets evaluated in this experiment were firstly screened by the *t*-test, and only the top-ranked 1000 features between the two groups of each dataset were loaded to the SI feature selection algorithms. Each SI algorithm selected features from the training dataset and evaluated these features on the validation dataset. The classification accuracy of the finally recommended features was calculated on the test dataset. For a fair comparison, the maximum number of iterations was set to 100 for all the five SI feature selection algorithms evaluated in this section.

GWO achieved the best averaged rank of 3.1429 for *N* = 10, as shown in Figure 4a. The prediction accuracies of the GWO-recommended features were ranked 7, 5, 2, 1, 2, 3 and 2 for the seven difficult datasets: ALL2, ALL3, ALL4, CNS, Colon, Mye and T1D, while the second-best averaged rank of 3.7143 was achieved by *N* = 30. From the perspective of prediction accuracies, GWO recommended the best averaged prediction accuracy of 0.7021 for the seven datasets when *N* = 10. The second-best averaged prediction accuracy was 0.6910 for *N* = 80. Thus, the following sections used *N* = 10 for the GWO algorithm.

WOA achieved the best averaged rank of 1.5714 on the seven datasets for *N* = 10, as shown in Figure 4b. The data showed that the WOA-selected features with *N* = 10 achieved the best prediction accuracies on four out of the seven datasets, i.e., ALL4, CNS, Mye and T1D. Although the WOA-selected features with *N* = 60 achieved a slightly better averaged accuracy of 0.6928 than that (averaged accuracy 0.6899) with *N* = 10, *N* = 60 only achieved the third-best averaged rank over the seven datasets. Thus, WOA used *N* = 10 in the following sections.

The FA-selected features achieved the best averaged rank for *N* = 30 and 50, as shown in Figure 4c. *N* = 70 achieved the best averaged accuracy of 0.7000, which was only slightly better than that (0.6861) for *N* = 30 and 50. A larger population size (*N*) required a longer running time. Thus, this study set *N* = 30 as the default population size for the FA feature selection algorithm.

MFO recommended features with *N* = 90 to achieve the best averaged rank (1.2857) and the best prediction accuracy (0.7076), as shown in Figure 4d. Actually, the MFO-selected features achieved the best prediction accuracies on six out of the seven evaluated datasets. Thus, the population size of MFO was set as 90 by default in this study.

Figure 4e shows that MRFO recommended the features achieving the best averaged rank (3.2857) and the best averaged prediction accuracy (0.6877) with *N* = 10. The second-best averaged rank (4.2857) was achieved with *N* = 100. Thus, the remainder of this study set the default population size *N* = 10 for MRFO.

### 3.3. Parameter Tunings of the Other Four SI Algorithms

The other four SI feature selection algorithms carried different parameters and were optimized separately. Due to the high complexities in the various parameters of these SI algorithms, the population size *N* and the number of iterations T were initialized as *N* = 50 and T = 100.

The Bat Algorithm (BA) had three parameters: pulse emission rate (R), loudness (A) and population size (N), which are evaluated in Figure S1a. To simplify the evaluation procedure, this study assumed R = A. Figure S1a shows that R = A = 0.8 achieved the best averaged rank of 1.2857 for the BA algorithm, and 474.00 were recommended by BA on average. Since R = A = 0.2 achieved a slightly worse averaged rank of 1.4286 with a better averaged number of features (460.14), this study chose R = A = 0.2 as the default value for BA. Then, the BA algorithm was evaluated for its different population sizes. Both the best averaged accuracy (0.6995) and the best averaged rank (1.4286) were achieved by *N* = 30 for the BA algorithm. Thus, the default population size N was set as 30.

The Particle Swarm Optimization (PSO) algorithm needed to set the lower bound of the inertia weight (denoted as MinW), which is evaluated in Figure S1b. The population size (N) was also evaluated. Both MinW = 0.1 and 0.2 achieved the best averaged accuracy of 0.6957 and the best averaged rank of 1.2857. However, the PSO algorithm recommended more than 27 features using MinW = 0.2 than when using MinW = 0.1. Thus, the remaining sections of this study set MinW = 0.1 as the default value. The PSO-selected features achieved the overall highest accuracy of 0.9500 using *N* = 80 on the dataset Colon, which was at least 0.1000 larger than the second-best accuracy of 0.8500. The averaged rank by *N* = 90 was 2.2857, the fourth-best averaged rank. This was mainly due to *N* = 80 achieving the accuracy of 0.6000 on the dataset CNS, which was smaller than that (0.6500) of the cases *N* = 40 and 90 with the best averaged rank of 2.0000. Thus, this study set *N* = 80 as the default population size of the PSO algorithm.

The Cuckoo Search (CS) algorithm mimicked the cuckoo’s reproduction behaviors, being found by the host birds [65,66]. The CS’s parameters ProbF and the population size (N) are evaluated in Figure S1c. The parameter ProbF = 0.8 achieved the best averaged rank of 1.8571 and the best averaged accuracy of 0.6933. Another value, ProbF = 0.3, achieved the second-best in both the averaged rank (2.2857) and the averaged accuracy (0.6861). Since the value ProbF = 0.3 was closer to the popular value choice of 0.25 [65,66] and recommended 6.86 fewer features than ProbF = 0.8, this study set ProbF = 0.3 as the default choice. The population size *N* = 60 achieved the best averaged rank of 2.1429, while the value *N* = 80 achieved the best averaged accuracy of 0.6869. This was due to the four values (*N* = 30, 50, 60, and 90) achieving the best accuracy of 0.8103, while *N* = 80 achieved a slightly worse accuracy of 0.7931. Since *N* = 80 achieved 0.0500 accuracy improvements on the two other datasets CNS and Colon, this study set *N* = 80 as the default population size of the CS algorithm.

The lower bound of the inertia weight (denoted as MinW) and the population size (*N*) of the dragonfly (DF) algorithm are evaluated in Figure S1d. The parameter MinW = 0.5 and 0.9 achieved the top two best averaged ranks of 0.6670 and 0.6645, respectively. These two values also achieved the top two best averaged accuracies of 0.6670 and 0.6645, respectively. Although these two values of the parameter MinW were only slightly different, the DF algorithm with MinW = 0.5 selected 97.71 features on average, which was much fewer than 147.14) with MinW = 0.9. Thus, the default value of MinW was set as 0.5 in this study. The population size *N* = 30 achieved the best in both average rank (1.7143) and the averaged accuracy (0.6937). Thus, this value (30) was set as the default value of the population size of the DF algorithm.

### 3.4. Finding the Best Classifier for Zoo

**Figure 5:**
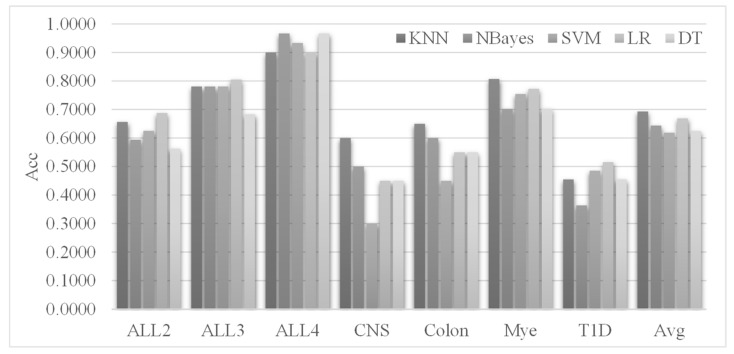
Performance comparison of the five classifiers on the features selected by the Zoo feature selection algorithm. The horizontal axis lists the seven datasets, and the last group of data columns gives the averaged performances of the five classifiers on the seven datasets. The vertical axis gives the prediction accuracies of the classifiers.

The Zoo-selected features were evaluated by five popular classifiers, i.e., KNN, NBayes, SVM, LR and DT, as shown in Figure 5. Each of the nine SI feature selection algorithms was executed for ten random runs on the training dataset, and the selected feature subset with the best prediction accuracy on the validation dataset was returned. The Zoo feature selection algorithm combined the nine feature subsets and carried out an additional feature screening using the dRFE algorithm to remove potentially redundant features [76]. The five classifiers evaluated the Zoo-selected features on the test dataset.

Figure 5 shows that the classifier KNN achieved the overall highest prediction accuracies on the seven datasets. Both KNN and LR achieved the best prediction accuracies on three datasets. It is interesting to observe that these two classifiers achieved the worst accuracy of 0.9000 on the ALL4 dataset, compared with the best accuracy of 0.9667 achieved by the NBayes and DT classifiers. Unfortunately, the NBayes and DT classifiers did not perform well on the other six datasets. This study recommends KNN as the default classifier to build prediction models using the Zoo-selected features.

### 3.5. Choosing the Maximum Number of Iterations

**Figure 6:**
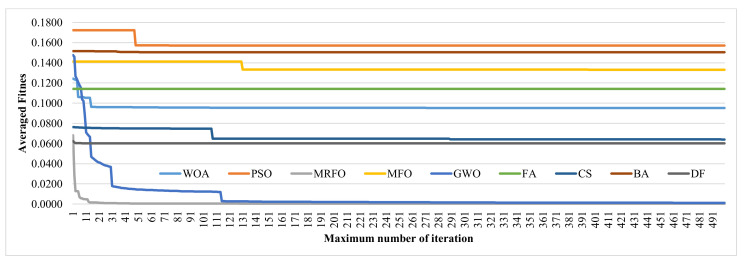
Evaluation of the maximum numbers of iterations for the nine SI feature selection algorithms. The horizontal axis lists the maximum numbers of iterations. The vertical axis gives the averaged fitness values of the selected feature subsets over the seven datasets.

We screened 500 iterations for the nine investigated SI feature selection algorithms, as shown in Figure 6. The curves in Figure 6 show that some SI algorithms converged to the minimum fitness values very early. The FA and DF algorithms converged at the first and eighth iterations, respectively. Figure 6 shows that all the SI feature selection algorithms reached stable averaged fitness values after 150 iterations. We evaluated the differences between the minimum fitness values within the total 500 iterations and the fitness on the 150th iteration. Besides FA and DF, the PSO algorithm also reached 0 in the difference. The BA algorithm achieved a difference of 4.29 × 10 ^−5^. The maximum difference of 1.2 × 10^−3^ was achieved by the GWO algorithm. Considering such minor differences in the fitness values and the time costs proportional to the numbers of iterations, this study chose the maximum number of iterations of T = 150 for all the nine SI feature selection algorithms.

### 3.6. Comparison with Other Feature Selection Algorithms

**Figure 7:**
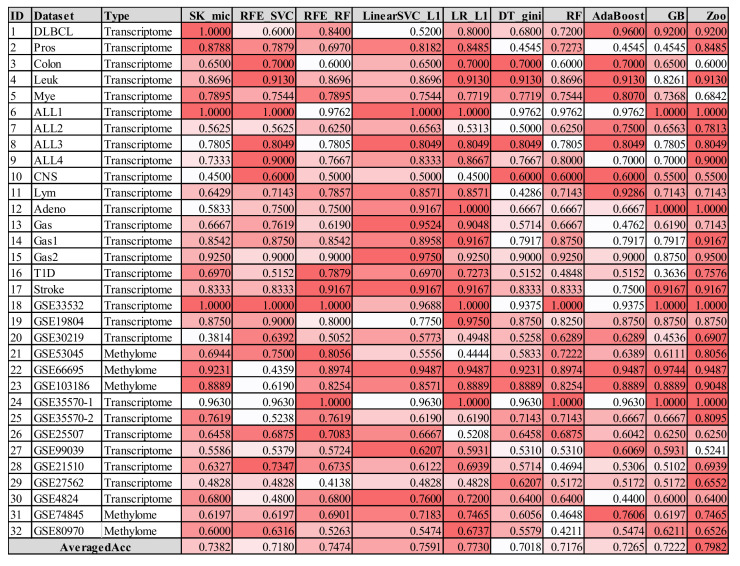
Heatmap table of the classification performances using the features recommended by the nine existing feature selection algorithms and Zoo. All of the 32 datasets were evaluated using the KNN classifier. Each row is the data of one dataset, and the last row is the averaged accuracy of each feature selection algorithms on the 32 datasets. A darker background represents a smaller accuracy in that row, and a white background represents the best accuracy in the same row. All the nine feature selection algorithms compared with Zoo were available as functions in the Python package sklearn version 0.19.2, as shown in Table S2.

The features selected by Zoo achieved generally satisfactory prediction accuracies for the 32 transcriptome and methylome datasets, as shown in Figure 7. Firstly, the Zoo-recommended features achieved the best averaged accuracy of 0.7982 for the 32 datasets. The feature selection algorithm LR_L1 achieved the second best averaged accuracy of 0.7730, while all the other eight feature selection algorithms did not achieve averaged accuracies better than 0.7600. Secondly, the Zoo-recommended features also achieved the best averaged rank of 2.7813 on the 32 datasets, and were ranked the best on 15 out of the 32 datasets.

The experimental data showed that the proposed feature selection algorithm Zoo tended to select features with very promising prediction accuracies compared with the nine existing algorithms.

## 4. Conclusions

This study proposed a novel feature selection algorithm, the Zoo algorithm, by integrating nine SI-based feature selection algorithms. Seven transcriptome datasets with small prediction accuracies in a previous study were used to tune the parameters of Zoo. The experimental data analysis showed that the SI-based feature selection algorithms recommended features with complementary contributions to each other, and their union needed an additional step of redundancy removal by feature selection algorithms such as dRFE. The comparison with the nine existing feature selection algorithms showed that the Zoo-recommended features achieved promising prediction accuracies on transcriptomics and methylomics datasets. It is recommended that the Zoo algorithm be combined with a KNN classifier to predict the performance of the selected feature subset.

The main limitation of Zoo was that the operating time is usually several hours due to the high time complexities of the SI-based feature selection algorithms. Additionally, the current version of Zoo did not efficiently integrate the internal operators of the nine SI feature selection algorithms.

## Data Availability

This study analyzed publicly available datasets, sources of which are detailed in Summary of Datasets. The proposed Zoo program is publicly available at http://www.healthinformaticslab.org/supp/resources.php.

## Supplementary Materials

The following are available online at https://www.mdpi.com/article/10.3390/genes12111814/s1, Table S1: Datasets evaluated in this study, Table S2: Details of the nine feature selection algorithms, Table S3: Default values for the parameters of the nine SI feature selection algorithms integrated in the Zoo algorithm, Figure S1: Optimizing the parameters of the remaining four SI feature selection algorithms.

## References

[1] D’Amelio S., Lombardo F., Pizzarelli A., Bellini I., Cavallero S. (2020). Advances in Omic Studies Drive Discoveries in the Biology of Anisakid Nematodes. Genes.

[2] Karczewski K.J., Snyder M.P. (2018). Integrative omics for health and disease. Nat. Rev. Genet..

[3] Liao J.G., Chin K.-V. (2007). Logistic regression for disease classification using microarray data: Model selection in a large p and small n case. Bioinformatics.

[4] Wu W., Ma X. (2020). Joint learning dimension reduction and clustering of single-cell RNA-sequencing data. Bioinformatics.

[5] Gao S., Wang P., Feng Y., Xie X., Duan M., Fan Y., Liu S., Huang L., Zhou F. (2021). RIFS2D: A two-dimensional version of a randomly restarted incremental feature selection algorithm with an application for detecting low-ranked biomarkers. Comput. Biol. Med..

[6] Wei Z., Ding S., Duan M., Liu S., Huang L., Zhou F. (2020). FeSTwo, a two-step feature selection algorithm based on feature engineering and sampling for the chronological age regression problem. Comput. Biol. Med..

[7] Wang Q., Lu Y., Zhang X., Hahn J. (2021). Region of Interest Selection for Functional Features. Neurocomputing.

[8] Chiesa M., Maioli G., Colombo G.I., Piacentini L. (2020). GARS: Genetic Algorithm for the identification of a Robust Subset of features in high-dimensional datasets. BMC Bioinform..

[9] Liu X., Zhang Y., Fu C., Zhang R., Zhou F. (2021). EnRank: An Ensemble Method to Detect Pulmonary Hypertension Biomarkers Based on Feature Selection and Machine Learning Models. Front. Genet..

[10] Li F., Yang M., Li Y., Zhang M., Wang W., Yuan D., Tang D. (2020). An improved clear cell renal cell carcinoma stage prediction model based on gene sets. BMC Bioinform..

[11] Sreejith S., Khanna Nehemiah H., Kannan A. (2020). Clinical data classification using an enhanced SMOTE and chaotic evolutionary feature selection. Comput. Biol. Med..

[12] Sahebi G., Movahedi P., Ebrahimi M., Pahikkala T., Plosila J., Tenhunen H. (2020). GeFeS: A generalized wrapper feature selection approach for optimizing classification performance. Comput. Biol. Med..

[13] Ding X., Yang F., Jin S., Cao J. (2021). An efficient alpha seeding method for optimized extreme learning machine-based feature selection algorithm. Comput. Biol. Med..

[14] Chalakkal R., Hafiz F., Abdulla W., Swain A. (2021). An efficient framework for automated screening of Clinically Significant Macular Edema. Comput. Biol. Med..

[15] Phadikar S., Sinha N., Ghosh R. (2021). Automatic Eyeblink Artifact Removal From EEG Signal Using Wavelet Transform With Heuristically Optimized Threshold. IEEE J. Biomed. Health Inform..

[16] Abu Khurmaa R., Aljarah I., Sharieh A. (2020). An intelligent feature selection approach based on moth flame optimization for medical diagnosis. Neural Comput. Appl..

[17] Liu W., Zhang M., Luo Z., Cai Y. (2017). An ensemble deep learning method for vehicle type classification on visual traffic surveillance sensors. IEEE Access.

[18] Abd El Aziz M., Hassanien A.E. (2018). Modified cuckoo search algorithm with rough sets for feature selection. Neural Comput. Appl..

[19] Mirjalili S., Lewis A. (2016). The Whale Optimization Algorithm. Adv. Eng. Softw..

[20] Yang X.-S., Gonzalez J.R., Pelta D.A., Cruz C., Terrazas G., Krasnogor N. (2010). A New metaheuristic bat-inspired algorithm. Nicso 2010: Nature Inspired Cooperative Strategies for Optimization.

[21] Yang X.-S. (2010). Firefly algorithm, stochastic test functions and design optimisation. Int. J. Bio-Inspired Comput..

[22] Mirjalili S. (2015). Moth-flame optimization algorithm: A novel nature-inspired heuristic paradigm. Knowl. -Based Syst..

[23] Binh T., Zhang M., Xue B. (2016). A PSO based hybrid feature selection algorithm for high-dimensional classification. 2016 IEEE Congress on Evolutionary Computation.

[24] Eberhart R., Kennedy J. A new optimizer using particle swarm theory. Proceedings of the MHS’95, 6th International Symposium on Micro Machine and Human Science.

[25] Zhao W., Zhang Z., Wang L. (2020). Manta ray foraging optimization: An effective bio-inspired optimizer for engineering applications. Eng. Appl. Artif. Intell..

[26] Mirjalili S. (2016). Dragonfly algorithm: A new meta-heuristic optimization technique for solving single-objective, discrete, and multi-objective problems. Neural Comput. Appl..

[27] Rostami M., Berahmand K., Nasiri E., Forouzandeh S. (2021). Review of swarm intelligence-based feature selection methods. Eng. Appl. Artif. Intell..

[28] Brezocnik L., Fister I., Podgorelec V. (2018). Swarm Intelligence Algorithms for Feature Selection: A Review. Appl. Sci..

[29] Han Y., Huang L., Zhou F. (2021). A dynamic recursive feature elimination framework (dRFE) to further refine a set of OMIC biomarkers. Bioinformatics.

[30] Ge R., Zhou M., Luo Y., Meng Q., Mai G., Ma D., Wang G., Zhou F. (2016). McTwo: A two-step feature selection algorithm based on maximal information coefficient. BMC Bioinform..

[31] Tian E., Zhan F.H., Walker R., Rasmussen E., Ma Y.P., Barlogie B., Shaughnessy J.D. (2003). The role of the Wnt-signaling antagonist DKK1 in the development of osteolytic lesions in multiple myeloma. N. Engl. J. Med..

[32] Wu Y., Grabsch H., Ivanova T., Tan I.B., Murray J., Ooi C.H., Wright A.I., West N.P., Hutchins G.G.A., Wu J. (2013). Comprehensive genomic meta-analysis identifies intra-tumoural stroma as a predictor of survival in patients with gastric cancer. Gut.

[33] Wang G., Hu N., Yang H.H., Wang L., Su H., Wang C., Clifford R., Dawsey E.M., Li J.-M., Ding T. (2013). Comparison of Global Gene Expression of Gastric Cardia and Noncardia Cancers from a High-Risk Population in China. PLoS ONE.

[34] Levy H., Wang X., Kaldunski M., Jia S., Kramer J., Pavletich S.J., Reske M., Gessel T., Yassai M., Quasney M.W. (2012). Transcriptional signatures as a disease-specific and predictive inflammatory biomarker for type 1 diabetes. Genes Immun..

[35] Krug T., Gabriel J.P., Taipa R., Fonseca B.V., Domingues-Montanari S., Fernandez-Cadenas I., Manso H., Gouveia L.O., Sobral J., Albergaria I. (2012). TTC7B emerges as a novel risk factor for ischemic stroke through the convergence of several genome-wide approaches. J. Cereb. Blood Flow Metab..

[36] Shipp M.A., Ross K.N., Tamayo P., Weng A.P., Kutok J.L., Aguiar R.C.T., Gaasenbeek M., Angelo M., Reich M., Pinkus G.S. (2002). Diffuse large B-cell lymphoma outcome prediction by gene-expression profiling and supervised machine learning. Nat. Med..

[37] Singh D., Febbo P.G., Ross K., Jackson D.G., Manola J., Ladd C., Tamayo P., Renshaw A.A., D’Amico A.V., Richie J.P. (2002). Gene expression correlates of clinical prostate cancer behavior. Cancer Cell.

[38] Chiaretti S., Li X.C., Gentleman R., Vitale A., Vignetti M., Mandelli F., Ritz J., Foa R. (2004). Gene expression profile of adult T-cell acute lymphocytic leukemia identifies distinct subsets of patients with different response to therapy and survival. Blood.

[39] Pomeroy S.L., Tamayo P., Gaasenbeek M., Sturla L.M., Angelo M., McLaughlin M.E., Kim J.Y.H., Goumnerova L.C., Black P.M., Lau C. (2002). Prediction of central nervous system embryonal tumour outcome based on gene expression. Nature.

[40] Alizadeh A.A., Eisen M.B., Davis R.E., Ma C., Lossos I.S., Rosenwald A., Boldrick J.G., Sabet H., Tran T., Yu X. (2000). Distinct types of diffuse large B-cell lymphoma identified by gene expression profiling. Nature.

[41] Notterman D.A., Alon U., Sierk A.J., Levine A.J. (2001). Transcriptional gene expression profiles of colorectal adenoma, adenocarcinoma, and normal tissue examined by oligonucleotide arrays. Cancer Res..

[42] Alon U., Barkai N., Notterman D.A., Gish K., Ybarra S., Mack D., Levine A.J. (1999). Broad patterns of gene expression revealed by clustering analysis of tumor and normal colon tissues probed by oligonucleotide arrays. Proc. Natl. Acad. Sci. USA.

[43] Golub T.R., Slonim D.K., Tamayo P., Huard C., Gaasenbeek M., Mesirov J.P., Coller H., Loh M.L., Downing J.R., Caligiuri M.A. (1999). Molecular classification of cancer: Class discovery and class prediction by gene expression monitoring. Science.

[44] Clough E., Barrett T. (2016). The Gene Expression Omnibus Database. Methods Mol. Biol..

[45] Alter M.D., Kharkar R., Ramsey K.E., Craig D.W., Melmed R.D., Grebe T.A., Bay R.C., Ober-Reynolds S., Kirwan J., Jones J.J. (2011). Autism and Increased Paternal Age Related Changes in Global Levels of Gene Expression Regulation. PLoS ONE.

[46] Shamir R., Klein C., Amar D., Vollstedt E.-J., Bonin M., Usenovic M., Wong Y.C., Maver A., Poths S., Safer H. (2017). Analysis of blood-based gene expression in idiopathic Parkinson disease. Neurology.

[47] Tsukamoto S., Ishikawa T., Iida S., Ishiguro M., Mogushi K., Mizushima H., Uetake H., Tanaka H., Sugihara K. (2011). Clinical Significance of Osteoprotegerin Expression in Human Colorectal Cancer. Clin. Cancer Res..

[48] Michael Meister A.B., Xu E.C., Schnabel P., Warth A., Hoffmann H., Dienemann H., Riedlinger J., Bodenmueller H., Zolg W., Herth F.J.F. (2014). Intra-tumor Heterogeneity of Gene Expression Profiles in Early Stage Non-Small Cell Lung Cancer. J. Bioinform. Res. Stud..

[49] Lu T.-P., Tsai M.-H., Lee J.-M., Hsu C.-P., Chen P.-C., Lin C.-W., Shih J.-Y., Yang P.-C., Hsiao C.K., Lai L.-C. (2010). Identification of a Novel Biomarker, SEMA5A, for Non-Small Cell Lung Carcinoma in Nonsmoking Women. Cancer Epidemiol. Biomark. Prev..

[50] LaBreche H.G., Nevins J.R., Huang E. (2011). Integrating Factor Analysis and a Transgenic Mouse Model to Reveal a Peripheral Blood Predictor of Breast Tumors. BMC Med. Genom..

[51] Rousseaux S., Debernardi A., Jacquiau B., Vitte A.-L., Vesin A., Nagy-Mignotte H., Moro-Sibilot D., Brichon P.-Y., Lantuejoul S., Hainaut P. (2013). Ectopic Activation of Germline and Placental Genes Identifies Aggressive Metastasis-Prone Lung Cancers. Sci. Transl. Med..

[52] Lockwood W.W., Chari R., Coe B.P., Girard L., MacAulay C., Lam S., Gazdar A.F., Minna J.D., Lam W.L. (2008). DNA amplification is a ubiquitous mechanism of oncogene activation in lung and other cancers. Oncogene.

[53] Dogan M.V., Shields B., Cutrona C., Gao L., Gibbons F.X., Simons R., Monick M., Brody G.H., Tan K., Beach S.R. (2014). The effect of smoking on DNA methylation of peripheral blood mononuclear cells from African American women. BMC Genom..

[54] Jones L.R., Young W., Divine G., Datta I., Chen K.M., Ozog D., Worsham M.J. (2015). Genome-Wide Scan for Methylation Profiles in Keloids. Dis Markers.

[55] Bartlett T.E., Chindera K., McDermott J., Breeze C.E., Cooke W.R., Jones A., Reisel D., Karegodar S.T., Arora R., Beck S. (2016). Epigenetic reprogramming of fallopian tube fimbriae in BRCA mutation carriers defines early ovarian cancer evolution. Nat. Commun..

[56] Smith R.G., Hannon E., de Jager P.L., Chibnik L., Lott S.J., Condliffe D., Smith A.R., Haroutunian V., Troakes C., Al-Sarraj S. (2018). Elevated DNA methylation across a 48-kb region spanning the HOXA gene cluster is associated with Alzheimer’s disease neuropathology. Alzheimers Dement..

[57] Huang K.K., Ramnarayanan K., Zhu F., Srivastava S., Xu C., Tan A.L.K., Lee M., Tay S., Das K., Xing M. (2018). Genomic and Epigenomic Profiling of High-Risk Intestinal Metaplasia Reveals Molecular Determinants of Progression to Gastric Cancer. Cancer Cell.

[58] Feng X., Li J., Li H., Chen H., Li F., Liu Q., You Z.-H., Zhou F. (2019). Age Is Important for the Early-Stage Detection of Breast Cancer on Both Transcriptomic and Methylomic Biomarkers. Front. Genet..

[59] Lombardo E., Kurz C., Marschner S., Avanzo M., Gagliardi V., Fanetti G., Franchin G., Stancanello J., Corradini S., Niyazi M. (2021). Distant metastasis time to event analysis with CNNs in independent head and neck cancer cohorts. Sci. Rep..

[60] Hichem H., Elkamel M., Rafik M., Mesaaoud M.T., Ouahiba C. (2019). A new binary grasshopper optimization algorithm for feature selection problem. J. King Saud. Univ..

[61] Zamani H., Nadimi-Shahraki M.-H. (2016). Feature selection based on whale optimization algorithm for diseases diagnosis. Int. J. Comput. Sci. Inf. Secur..

[62] Nakamura R.Y., Pereira L.A., Costa K.A., Rodrigues D., Papa J.P., Yang X.-S. BBA: A binary bat algorithm for feature selection. Proceedings of the 2012 25th SIBGRAPI Conference on Graphics, Patterns and Images.

[63] Yang X.-S., Deb S. Cuckoo search via Lévy flights. Proceedings of the 2009 World Congress on Nature & Biologically Inspired Computing (NaBIC).

[64] Rodrigues D., Pereira L.A., Almeida T., Papa J.P., Souza A., Ramos C.C., Yang X.-S. BCS: A binary cuckoo search algorithm for feature selection. Proceedings of the 2013 IEEE International Symposium on Circuits and Systems (ISCAS).

[65] Yang X.-S. (2008). Nature-Inspired Metaheuristic Algorithms.

[66] Yang X.-S. Firefly algorithms for multimodal optimization. Proceedings of the International Symposium on Stochastic Algorithms.

[67] Zawbaa H.M., Emary E., Parv B., Sharawi M. Feature selection approach based on moth-flame optimization algorithm. Proceedings of the 2016 IEEE Congress on Evolutionary Computation (CEC).

[68] Kennedy J., Eberhart R. Particle swarm optimization. Proceedings of the ICNN’95-International Conference on Neural Networks.

[69] Sharkawy R., Ibrahim K., Salama M., Bartnikas R. (2011). Particle swarm optimization feature selection for the classification of conducting particles in transformer oil. IEEE Trans. Dielectr. Electr. Insul..

[70] Ghosh K.K., Guha R., Bera S.K., Kumar N., Sarkar R. (2021). S-shaped versus V-shaped transfer functions for binary Manta ray foraging optimization in feature selection problem. Neural Comput. Appl..

[71] Mafarja M.M., Eleyan D., Jaber I., Hammouri A., Mirjalili S. Binary dragonfly algorithm for feature selection. Proceedings of the 2017 International Conference on New Trends in Computing Sciences (ICTCS).

[72] Mirjalili S., Mirjalili S.M., Lewis A. (2014). Grey Wolf Optimizer. Adv. Eng. Softw..

[73] Emary E., Zawbaa H.M., Hassanien A.E. (2016). Binary grey wolf optimization approaches for feature selection. Neurocomputing.

[74] Yang X.-S. (2020). Nature-inspired optimization algorithms: Challenges and open problems. J. Comput. Sci..

[75] Khurma R.A., Aljarah I., Sharieh A., Mirjalili S., Mirjalili S., Faris H., Aljarah I. (2020). EvoloPy-FS: An open-source nature-inspired optimization framework in python for feature selection. Evolutionary Machine Learning Techniques: Algorithms and Applications.

[76] Faris H., Aljarah I., Mirjalili S., Castillo P.A., Merelo J.J. (2016). EvoloPy: An Open-Source Nature-Inspired Optimization Framework in Python.

[77] Abdel-Basset M., El-Shahat D., El-henawy I., de Albuquerque V.H.C., Mirjalili S. (2020). A new fusion of grey wolf optimizer algorithm with a two-phase mutation for feature selection. Expert Syst. Appl..

[78] Ye Y., Zhang R., Zheng W., Liu S., Zhou F. (2017). RIFS: A randomly restarted incremental feature selection algorithm. Sci. Rep..

