# Supplementary material for: Zoo: Selecting Transcriptomic and Methylomic Biomarkers by Ensembling Animal-Inspired Swarm Intelligence Feature Selection Algorithms
Source: Genes (Basel). 2021 Nov 18;12(11):1814. doi: 10.3390/genes12111814 (PMC8621246; doi:10.3390/genes12111814)
Supplement: Supplementary file 1 [file genes-12-01814-s001.zip › genes-1352038-supplementary-done.pdf]

genes

MDPI

Zoo, Selecting Transcriptomic and Methylomic Biomarkers by Ensembling Animal-Inspired Swarm Intelligence Feature Selection Algorithms

Yuanyuan Han, Lan Huang * and Fengfeng Zhou*

College of Computer Science and Technology, and Key Laboratory of Symbolic Computation and Knowledge Engineering of Ministry of Education, Jilin University, Changchun, Jilin, China, 130012.

* Correspondence: FengfengZhou@gmail.com or ffzhou@jlu.edu.cn (F.Z.).

Table S1

Datasets evaluated in this study. The column "Dataset" gave the abbreviation of each dataset. Two types of datasets were used in this study, i.e., Transcriptome and Methylome, as described in the column "Type". The column "Phenotype" gave the full names of the investigated phenotypes. The detailed information of the two classes of the samples were given in the column "Classes", with the bracketed sample numbers for each class. The numbers of total features and samples were listed in the column "FNum" and "SNum".

ID	Dataset	Type	Phenotype	Classes	FNum	SNum
1	DLBCL	Transcriptome	diffuse large B-cell lymphoma	DLBCL patients (58) and follicular lymphoma (19)	7,129	77
2	Pros	Transcriptome	prostate cancer	prostate (52) and non-prostate (50)	12,625	102
3	Colon	Transcriptome	colon cancer	tumour (40) and normal (22)	2,000	62
4	Leuk	Transcriptome	acute lymphocytic leukemia vs acute myeloid leukemia	ALL (47) and AML (25)	7,129	72
5	Mye	Transcriptome	myeloma	presence (137) and absence (36) of focallesions of bone	12,625	173

6	ALL1	Transcriptome	acute lymphocytic leukemia	B-cell (95) and T-cell (33)	12,625	128
7	ALL2	Transcriptome	acute lymphocytic leukemia	Patients that did (65) and did not (35) relapse	12,625	100
8	ALL3	Transcriptome	acute lymphocytic leukemia	with (24) and without (101) multidrug resistance	12,625	125
9	ALL4	Transcriptome	acute lymphocytic leukemia	with (26) and without (67) the t(9;22) chromosome translocation	12,625	93
10	CNS	Transcriptome	central nervous system tumor	medulloblastoma survivors (39) and treatment failures (21)	7,129	60
11	Lym	Transcriptome	diffuse large B-cell lymphoma	germinalcentre (22) and activated B-like DLBCL (23)	4,026	45
12	Adeno	Transcriptome	colon adenocarcinoma	colon adenocarcinoma (18) and normal (18)	7,457	36
13	Gas	Transcriptome	gastric cancer	tumors (29) and non-malignants (36)	22,645	65
14	Gas1	Transcriptome	non-cardia gastric cancer	non-cardia (72) of gastric and normal (72)	22,283	144
15	Gas2	Transcriptome	cardia gastric cancer	cardia (62) of gastric and normal (62)	22,283	124
16	T1D	Transcriptome	type 1 diabetes	T1D (57) and healthy control (44)	54,675	101
17	Stroke	Transcriptome	ischemic stroke	ischemic stroke (20) and control (20)	54,675	40
18	GSE33532	Transcriptome	lung cancer	primary lung cancers (80) and distant unaffected lung tissue (20)	54,675	100
19	GSE19804	Transcriptome	lung cancer	60 paired lung cancers and adjacent normal lung tissue	12,625	120

20	GSE30219	Transcriptome	lung cancer	early-stage (N0) lung cancers (198) and lung cancers on the other stages (N1, N2, or N3)(93)	12,625	291
21	GSE35570-2	Transcriptome	PTC samples with the radiation treatments	PTC samples with the radiation treatments(33) and PTC samples without the radiation treatments(32)	54,675	65
22	GSE25507	Transcriptome	pediatric autism	peripheral blood lymphocytes (PBL) of pediatric autism patients (82) and healthy children(64)	54,675	146
23	GSE99039	Transcriptome	Idiopathic Parkinson's disease	patients (205)and controls(233)	54,675	438
24	GSE21510	Transcriptome	colorectal cancer	metastatic recurrent colorectal cancers (54) and primary colorectal cancers(94)	54,675	148
25	GSE27562	Transcriptome	breast cancer	invasive breast cancer patients (51) and patients with benign diagnosis (37)	12,625	88
26	GSE4824	Transcriptome	lung cancer	male(52) and female(25) lung cancer cell lines	22,283	77
27	GSE35570-1	Transcriptome	periodic thyroid cancer (PTC) without radiation treatment	periodic thyroid cancer (PTC) without radiation treatment(32) and normal samples(51)	54,675	83
28	GSE53045	Methylome	smoking	peripheral blood mononuclear cells (PBMC) for smokers (50)and non-smokers(61)	485,577	111
29	GSE66695	Methylome	breast cancer	breast cancer patients (80) and normal controls(40)	485,577	120
30	GSE103186	Methylome	gastric light or mild intestinal metaplasia	gastric light or mild intestinal metaplasia(130) and gastric normal controls(61)	467,971	191
31	GSE74845	Methylome	breast cancer	Fimbria(110) and proximal(106) tubal DNA samples	470,425	216
32	GSE80970	Methylome	Alzheimer’s Disease	Alzheimer’s Disease (148)samples and control(138) brain tissues	485,577	286

Table S2

Details of the nine feature selection algorithms. The column "Algorithm" gives the names of the feature selection algorithms. The column "Function" gives the library function of this algorithm in the Python package sklearn version 0.19.2 used in this study. The last column "Parameter" gives how this function is called for this feature selection algorithm.

Algorithm	Function	Parameter
DT_gini	DecisionTreeClassifier()	default parameters
RF	RandomForestClassifier()	default parameters
AdaBoost	AdaBoostClassifier()	default parameters
GB	GradientBoostingClassifier()	default parameters
LR_L1	penalty;solver	l1;liblinear
lSVC_L1	penalty;loss;dual	l1;squared_hinge;False
RFE_SVC	estimator;step	LinearSVC();0.5
RFE_RF	estimator;step	RandomForestClassifier();0.5
SK_mic	score_func	mutual_info_classif

Table S3

Default values for the parameters of the nine SI feature selection algorithms integrated in the Zoo algorithm.

Algorithm	Parameter	Value
CS	Pa	0.3
DF	Inertia weight lower limit	0.5
BA	Loudness A	0.2
BA	Pulse frequency r	0.2
PSO	Cognitive learning factor c1	2
PSO	Social learning factor c2	2
PSO	Inertia weight loss lower limit ωmin	0.1
CS	N	80
DF	N	30
BA	N	30
PSO	N	80
GWO	N	10
WOA	N	10
FA	N	30
MFO	N	90
MRFO	N	10
Nine SI algorithms	Max number of iterations (T)	150

Figure S1

Optimizing the parameters of the remaining four SI feature selection algorithms. (a) The parameters pulse emission rate (R), loudness (A) and population size (N) of the bat algorithm (BA) were evaluated by the data in the two heatmaps, assuming R=A for simplicity. (b) The lower bound of the inertia weight (MinW) and the population size (N) of the particle swarm optimization (PSO) algorithm were evaluated. (c) The probability of a cuckoo-laid egg being found by the host bird (ProbF) and the population size (N) of the cuckoo search (CS) algorithm were evaluated in the two heatmaps. And (d) the two heatmaps evaluated different value choices of the lower bound of the inertia weight (MinW) and the population size (N) of the dragonfly (DF) algorithm.

**R=A**	0.1	0.2	0.3	0.4	0.5	0.6	0.7	0.8	0.9
ALL2	0.5588	0.5588	0.5588	0.5588	0.5588	0.5588	0.5588	0.5588	0.5588
ALL3	0.7381	0.7619	0.7381	0.7619	0.7381	0.7381	0.7381	0.7381	0.7381
ALL4	0.7097	0.7097	0.7097	0.7097	0.7097	0.7097	0.7097	0.7097	0.7097
CNS	0.7000	0.7000	0.7000	0.6000	0.7000	0.7000	0.6000	0.7000	0.7000
Colon	0.7500	0.7500	0.8000	0.7500	0.8000	0.7500	0.7500	0.8000	0.7500
Mye	0.7931	0.8103	0.7931	0.7931	0.7931	0.7931	0.8103	0.8103	0.7931
T1D	0.5294	0.5294	0.5294	0.5294	0.5294	0.5294	0.5000	0.5294	0.5294

**N**	10	20	30	40	50	60	70	80	90	100
ALL2	0.5588	0.5588	0.5588	0.5588	0.5588	0.5588	0.5588	0.5588	0.5588	0.5588
ALL3	0.7619	0.7619	0.7381	0.7381	0.7381	0.7381	0.7381	0.7619	0.7381	0.7381
ALL4	0.7097	0.7097	0.7097	0.7097	0.7097	0.7097	0.7097	0.7097	0.7097	0.7097
CNS	0.6000	0.6000	0.7000	0.7000	0.7000	0.6000	0.7000	0.7000	0.6500	0.7000
Colon	0.7500	0.8500	0.8500	0.7500	0.8000	0.7500	0.8000	0.7500	0.7500	0.7500
Mye	0.7931	0.8103	0.8103	0.7931	0.7931	0.8103	0.7931	0.8103	0.8103	0.8103
T1D	0.4706	0.5294	0.5294	0.5294	0.5294	0.5294	0.5294	0.5294	0.5294	0.5294

(a)

**MinW**	0.1	0.2	0.3	0.4	0.5	0.6	0.7	0.8	0.9
ALL2	0.5588	0.5588	0.5588	0.5588	0.5588	0.5588	0.5588	0.5588	0.5588
ALL3	0.7619	0.7619	0.7619	0.7381	0.7619	0.7619	0.7381	0.7381	0.7381
ALL4	0.7097	0.7097	0.7097	0.7097	0.7097	0.7097	0.7097	0.7097	0.7097
CNS	0.6500	0.6500	0.6500	0.6500	0.7000	0.6500	0.7000	0.5500	0.6000
Colon	0.8500	0.8500	0.8000	0.7500	0.7500	0.8000	0.8500	0.8000	0.8500
Mye	0.8103	0.8103	0.7931	0.7931	0.7931	0.7931	0.7931	0.7931	0.7931
T1D	0.5294	0.5294	0.5294	0.5294	0.5294	0.5294	0.5000	0.5294	0.5294

**N**	10	20	30	40	50	60	70	80	90	100
ALL2	0.5588	0.5588	0.5588	0.5588	0.5588	0.5588	0.5588	0.5588	0.5588	0.5588
ALL3	0.7381	0.7381	0.7381	0.7619	0.7381	0.7381	0.7619	0.7381	0.7619	0.7381
ALL4	0.7097	0.7097	0.7097	0.7097	0.7097	0.7097	0.7097	0.7097	0.7097	0.7097
CNS	0.7000	0.6500	0.6000	0.6500	0.7000	0.5500	0.5500	0.6000	0.6500	0.6000
Colon	0.8000	0.7500	0.7500	0.7500	0.7500	0.8000	0.8500	0.9500	0.7500	0.7500
Mye	0.7931	0.7931	0.8103	0.7931	0.7931	0.7931	0.7931	0.7931	0.7931	0.7931
T1D	0.5000	0.5294	0.5294	0.5294	0.5294	0.5294	0.5294	0.5294	0.5294	0.5294

(b)

**ProbF**	0.1	0.2	0.3	0.4	0.5	0.6	0.7	0.8	0.9
ALL2	0.5588	0.5588	0.5588	0.5588	0.5588	0.5588	0.5882	0.5588	0.5588
ALL3	0.7619	0.7619	0.7619	0.7381	0.7619	0.7381	0.7619	0.7619	0.7381
ALL4	0.7097	0.7097	0.7097	0.7097	0.7097	0.7097	0.7097	0.7097	0.7097
CNS	0.6000	0.6000	0.6500	0.6000	0.5000	0.5500	0.5500	0.6500	0.7000
Colon	0.9000	0.7500	0.8000	0.8500	0.8500	0.9000	0.8000	0.8500	0.8000
Mye	0.7931	0.8103	0.7931	0.7931	0.7931	0.8103	0.7931	0.7931	0.7931
T1D	0.4706	0.5000	0.5294	0.5294	0.5294	0.5000	0.5294	0.5294	0.5000

**N**	10	20	30	40	50	60	70	80	90	100
ALL2	0.5588	0.5588	0.5588	0.5588	0.5294	0.5588	0.5588	0.5882	0.5588	0.5588
ALL3	0.7381	0.7381	0.7619	0.7381	0.7619	0.7619	0.7619	0.7381	0.7619	0.7381
ALL4	0.7097	0.7097	0.7097	0.7097	0.7097	0.7097	0.7097	0.7097	0.7097	0.7097
CNS	0.6000	0.6000	0.6500	0.5500	0.5000	0.5500	0.5500	0.6000	0.6000	0.5500
Colon	0.8000	0.8000	0.7500	0.8000	0.8000	0.8000	0.8500	0.8500	0.7500	0.8000
Mye	0.7931	0.7931	0.8103	0.7931	0.8103	0.8103	0.7931	0.7931	0.8103	0.7931
T1D	0.5000	0.5294	0.5000	0.5294	0.5000	0.5294	0.5294	0.5294	0.5294	0.5294

(c)

**MinW**	0.1	0.2	0.3	0.4	0.5	0.6	0.7	0.8	0.9
ALL2	0.6176	0.5294	0.6176	0.5882	0.5588	0.5882	0.5588	0.5588	0.5588
ALL3	0.7619	0.7381	0.7143	0.7619	0.7619	0.7857	0.7619	0.7619	0.7619
ALL4	0.7097	0.7097	0.7419	0.7097	0.7419	0.7097	0.7419	0.7097	0.7097
CNS	0.6500	0.6500	0.4000	0.6000	0.7000	0.6000	0.6000	0.6500	0.7000
Colon	0.7759	0.7759	0.7759	0.7759	0.7931	0.7931	0.7759	0.7931	0.8103
Mye	0.5000	0.4412	0.5000	0.4412	0.5294	0.5000	0.4706	0.4706	0.5294
T1D	0.5736	0.5492	0.5357	0.5538	0.5836	0.5681	0.5584	0.5634	0.5815

**N**	10	20	30	40	50	60	70	80	90	100
ALL2	0.5588	0.5588	0.5588	0.5294	0.5588	0.5000	0.5588	0.5588	0.5882	0.5882
ALL3	0.7619	0.7619	0.7619	0.7143	0.7381	0.7619	0.7381	0.7381	0.7381	0.7619
ALL4	0.7419	0.6774	0.7419	0.6774	0.7097	0.7097	0.7419	0.7097	0.7419	0.7097
CNS	0.5000	0.6000	0.6500	0.5500	0.5500	0.6000	0.6000	0.5500	0.6000	0.5000
Colon	0.8000	0.8500	0.8500	0.8500	0.8000	0.7500	0.9000	0.8000	0.7000	0.7500
Mye	0.8103	0.7931	0.7931	0.7931	0.7759	0.7931	0.7931	0.7931	0.7931	0.7931
T1D	0.5000	0.5294	0.5000	0.5000	0.5000	0.5000	0.4412	0.5000	0.5000	0.4706

(d)
